# IGFBP6 is a novel nasopharyngeal carcinoma prognostic biomarker

**DOI:** 10.18632/oncotarget.11886

**Published:** 2016-09-07

**Authors:** Qiuyan Chen, Siyuan Qin, Yang Liu, Minghuang Hong, Chao-Nan Qian, Evan T. Keller, Jian Zhang, Yi Lu

**Affiliations:** ^1^ State Key Laboratory of Oncology in South China, Collaborative Innovation Center for Cancer Medicine, Sun Yat-sen University Cancer Center, Guangzhou, Guangdong, China; ^2^ Department of Nasopharyngeal Carcinoma, Sun Yat-sen University Cancer Center, Guangzhou, Guangdong, China; ^3^ Key Laboratory of Longevity and Aging-related Diseases, Ministry of Education, Guangxi Medical University, Nanning, Guangxi, China; ^4^ Center for Translational Medicine, Guangxi Medical University, Nanning, Guangxi, China; ^5^ Department of Urology and Pathology, University of Michigan School of Medicine, Ann Arbor, Michigan, USA; ^6^ Department of Pathology, University of Pittsburgh School of Medicine, Pittsburgh, Pennsylvania, USA

**Keywords:** IGFBP6, GSK3β, β-catenin, cyclin D1, nasopharyngeal carcinoma

## Abstract

Insulin-like growth factor binding proteins (IGFBPs) play critical roles in carcinogenesis. This study assessed the impact of IGFBP6 on the progression of nasopharyngeal carcinoma (NPC). Using immunohistochemical analysis, we found that IGFBP6 was differentially expressed in primary malignant NPC tissues. Clinical samples were divided into two groups: IGFBP6(+) and IGFBP6(−). Five years of follow-up revealed that overall survival and distant metastasis-free survival rates were significantly higher in the IGFBP6(+) than IGFBP6(−) group. We also used real-time PCR, ELISA and western blot assays to measure IGFBP6 levels in five NPC cell lines (CNE1, CNE2, HONE1, HK1 and SUNE1). All the cell lines expressed IGFBP6, but at different levels, reflecting disease heterogeneity. In addition, exogenous expression of IGFBP6 inhibited CNE2 cell proliferation and invasion *in vitro*. IGFBP6 knockdown activated the GSK3β/β-catenin/cyclin D1 pathway and enhanced CNE2 tumor cell growth and metastasis in a mouse model. These results suggest that IGFBP6 may be an independent prognostic biomarker for NPC.

## INTRODUCTION

Nasopharyngeal carcinoma (NPC) is endemic in southern China and Southeast Asia, but rare in European and American countries [1–4]. Radiotherapy is the main therapeutic option and chemotherapy is often used in advanced cases. Distant metastasis, most commonly to bone, lung or liver, is the main cause of treatment failure [5]. Distant metastases are observed in 5% of newly-diagnosed NPC cases and are estimated to occur in about 21.2–41.8% of patients within 5 years of radiotherapy [6]. The incidence of distant metastasis is higher in patients with advanced disease, and the molecular mechanisms of NPC progression and metastasis are still unclear.

The insulin-like growth factor (IGF) family regulates cellular proliferation, differentiation and apoptosis, as well as carcinogenesis [7]. These diverse biological activities are mediated primarily via IGF association with receptor types I and II (IGF-IR and IGF-IIR). IGF is in turn regulated by a group of high-affinity IGF-binding proteins (IGFBP1–6). Recent epidemiological studies suggested that IGFBPs are associated with increased risk for several common cancers [8]. IGFBP6, an O-glycosylated protein with a predicted molecular weight of 34 kDa [9], can inhibit cell proliferation through specific binding to IGF-II [10, 11]. As an IGF-II inhibitor, IGFBP6 expression is reduced in malignant cells [12]. IGFBP6 inhibits growth in a number of IGF-II-dependent cancers, including rhabdomyosarcoma [13], neuroblastoma [14], colon cancer [15, 16], lung cancer [17], prostate cancer [18], breast cancer [19, 20] and ovarian cancer [21]. Gene array studies also showed that IGFBP6 expression has an antiproliferative effect in cancer [22, 23]. IGFBP6 overexpression inhibits proliferation and promotes rhabdomyosarcoma cell apoptosis *in vitro* and dramatically inhibits xenograft growth *in vivo* [13].

Though IGFBP6 appears to act as an inhibitory agent in various tumors, some studies found that it stimulated growth in a smaller number of tumors [12]. In ovarian cancer cell lines, differential effects of IGFBP6 on migration were observed [24]. Increased IGFBP6 expression correlated with increasing degrees of brain tissue invasion by meningioma cells [25, 26]. Only one report proposed that IGFBP6 acts as a tumor suppressor gene in NPC [27], and the biological functions of IGFBP6 in NPC progression remain unclear. In the current study, we explored the effects of IGFBP6 on NPC progression and evaluated IGFBP6 as a potential independent NPC prognostic biomarker.

## RESULTS

### IGFBP6 is differentially expressed in NPC clinical specimens

**Figure 1:**
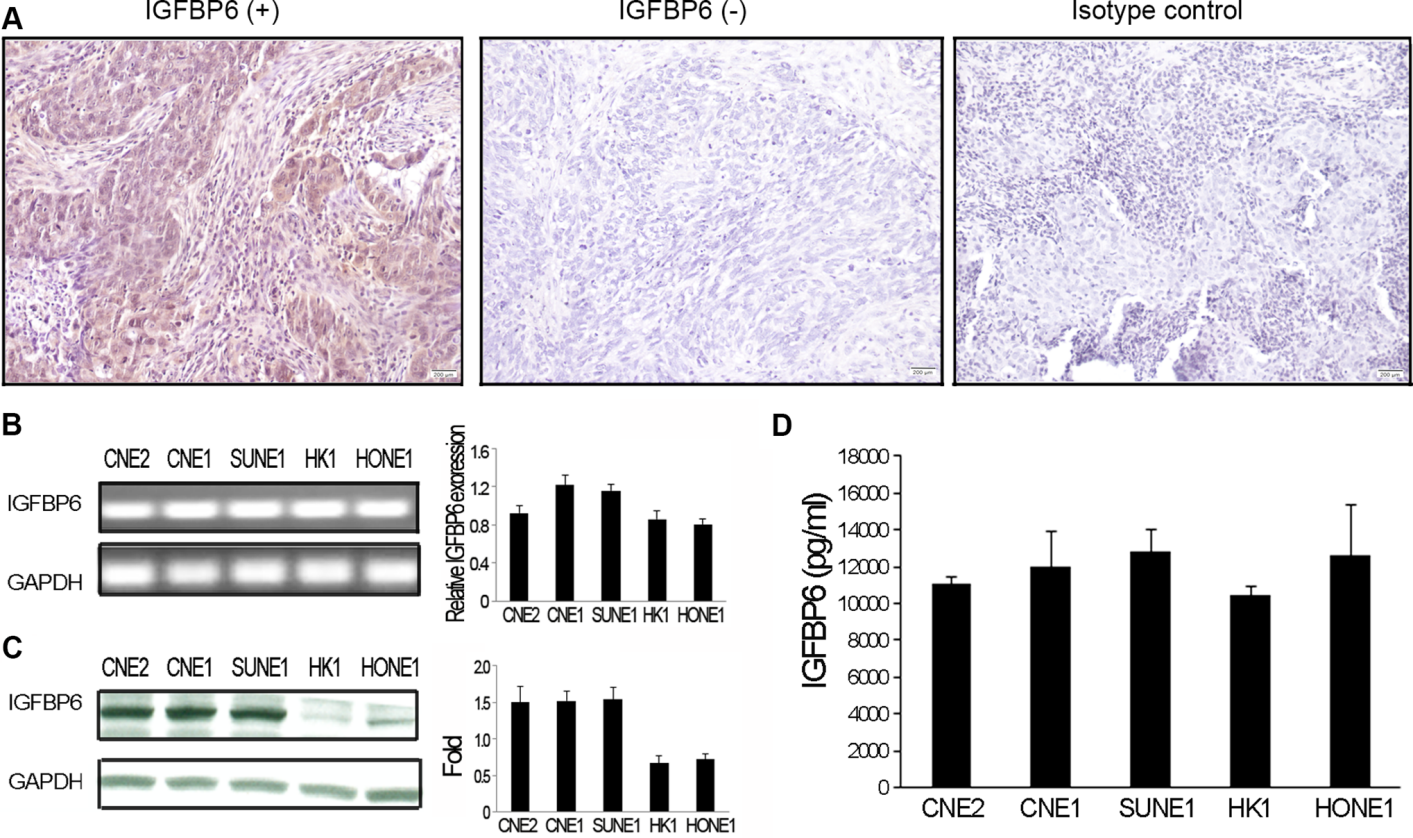
IGFBP6 expression in NPC clinical specimens and cell lines IGFBP6 was detected by immunostaining in primary NPC tissues (magnification ×200). Left:, IGFBP6 positive staining; Middle: IGFBP6 negative staining; Right: isotype control staining (**A**) IGFBP6 mRNA was measured in five NPC cell lines (CNE2, CNE1, SUNE1, HK1 and HONE1) via RT-PCR, with GAPDH as an internal control (**B**) Data are representative of three separate experiments. Western blotting of whole-cell lysates to detect IGFBP6 (**C**) IGFBP6 levels in CM from NPC cells as measured by ELISA (**D**) Data are representative of two separate experiments. All data represent means ± SD from triplicates.

We examined IGFBP6 protein levels in biopsy specimens from primary and advanced NPC patients. Seventy-six specimens were assessed from September 1998 to December 2004, including 58 male and 18 female cases. The median patient age was 47 years (range, 23–64). All specimens were graded using pathologic and clinical stage. Immunohistochemical (IHC) staining revealed differential IGFBP6 expression in NPC specimens. Positive cytoplasmic IGFBP6 staining was brown, and was not observed in negative or isotype controls (Figure 1A). Fifty-four out of 76 NPC cases (71.1%) exhibited positive IGFBP6 staining, and 22 (28.9%) were negative. No correlation was found between IGFBP6 expression and patient gender, age, T classification, N classification or clinical stage (Table 1).

**Table 1:** Correlation of IGFBP6 expression with clinical characteristics in patients with NPC

Characteristic	case 76	IGFBP6 expression (No.%)		*P* value
		Negative	Positive	
Age (y)				
≤ 46	38	10 (26.3)	28 (73.7)	0.613
> 46	38	12 (31.6)	26 (68.4)	
Gender				
Male	58	16 (27.6)	42 (72.4)	0.639
Female	18	6 (33.3)	12 (66.7)	
T classification				
T1–T2	16	8 (50.0)	8 (50.0)	0.075
T3–T4	60	14 (23.3)	46 (76.7)	
N classification				
N0–N1	26	8 (30.8)	18 (69.2)	0.801
N2–N3	50	14 (28.0)	36 (72.0)	
Clinical stage				
III	24	8 (33.3)	16 (66.7)	0.567
IV	52	14 (26.9)	38 (73.1)	

### NPC cells express IGFBP6

To verify IHC staining results, IGFBP6 mRNA levels were assessed in NPC cell lines (CNE2, CNE1, HONE1, HK1 and SUNE1) by RT-PCR and real-time PCR. IGFBP6 was expressed in all tested cells (Figure 1B). IGFBP6 protein levels were determined by western blot and enzyme linked immunosorbent assay (ELISA) in whole cell lysates or conditioned medium (CM) collected from NPC cell lines. All NPC lines expressed IGFBP6, but at different levels (Figure 1C–1D). We also found that IGFBP6 expression was reduced in primary HNEC cells (PromoCell GmbH, Sickingenstrasse, Germany) as compared to NPC cells (data not shown).

### IGFBP6 is an independent prognostic factor for locoregional relapse and distant metastasis

**Figure 2:**
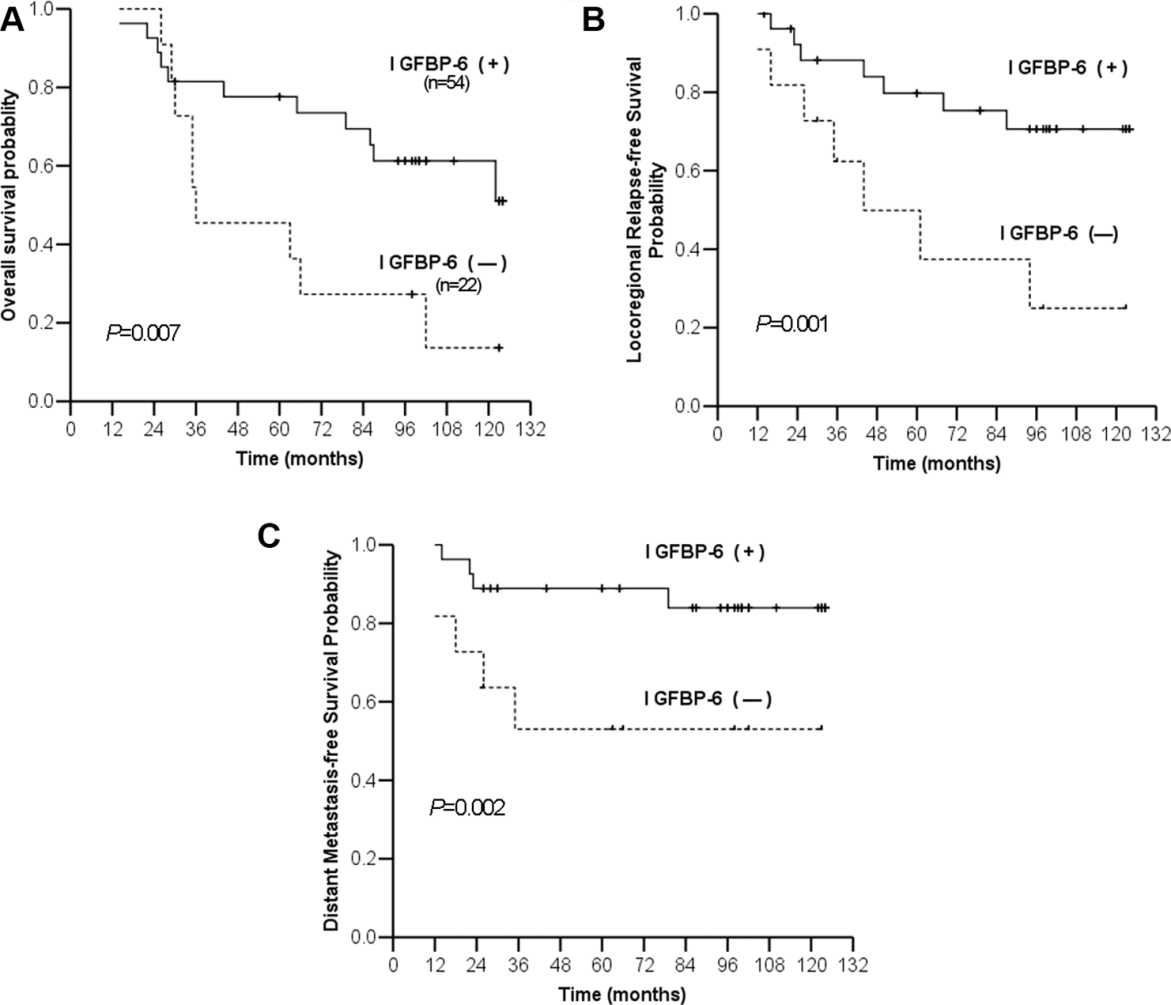
Kaplan-Meier estimates of survival curves for negative and positive IGFBP6 expression Overall survival (**A**) Locoregional relapse-free survival (**B**) Distant metastasis-free survival (**C**).

Up to December 2010, median follow-up time was 107.9 mo (range, 14–145). Among the 76 NPC patients, 28 developed locoregional relapse and 18 developed distant organ metastases (to bone, lung and/or liver). The five-year overall survival (OS) rate for all patients was 60% (46/76). Five-year OS rates in the IGFBP6 (−) and IGFBP6 (+) groups were 27% and 73%, respectively (*P* = 0.007). Five-year locoregional relapse-free survival (LRFS) rates were 38% and 75%, respectively (*P* = 0.001), and five-year distant metastasis-free survival (DMFS) rates were 53% and 89%, respectively (*P* = 0.002) (Figure 2). Multivariate analysis in the Cox model showed that IGFBP6 expression and patient clinical stage were independent prognostic factors for OS, LRFS and DMFS (Table 2). Positive IGFBP6 expression indicated lower risk of locoregional relapse and distant metastasis.

**Table 2:** Multivariate Cox regression analysis for survival prognostic factors in advanced nasopharyngeal carcinoma

End point	HR	95%CI	*P*
Overall survival			
Sex	0.47	0.19–1.17	0.10
Age	1.04	0.99–1.10	0.056
T stage	1.12	0.67–1.87	0.64
N stage	1.47	0.91–2.38	0.11
Clinical stage	0.34	0.15–0.79	0.012^*^
IGFBP6 expression	0.28	0.13–0.62	0.002^*^
Locoregional relapse-free survival			
Sex	0.45	0.15–1.36	0.15
Age	1.12	0.97–1.16	0.057
T stage	0.98	0.53–1.81	0.94
N stage	1.40	0.78–2.53	0.25
Clinical stage	0.33	0.12–0.91	0.033^*^
IGFBP6 expression	0.34	0.13–0.85	0.021^*^
Distant metastasis-free survival			
Sex	0.49	0.13–1.78	0.28
Age	0.97	0.91–1.03	0.36
T stage	1.06	0.51–2.21	0.87
N stage	0.99	0.49–2.00	0.98
Clinical stage	0.33	0.10–1.04	0.06
IGFBP6 expression	0.20	0.06–0.67	0.009^*^

Abbreviations: HR = hazard ratio; CI = confidence interval.

* *P* < 0.05.

### Exogenous IGFBP6 inhibits NPC cell growth and invasion *in vitro*

**Figure 3:**
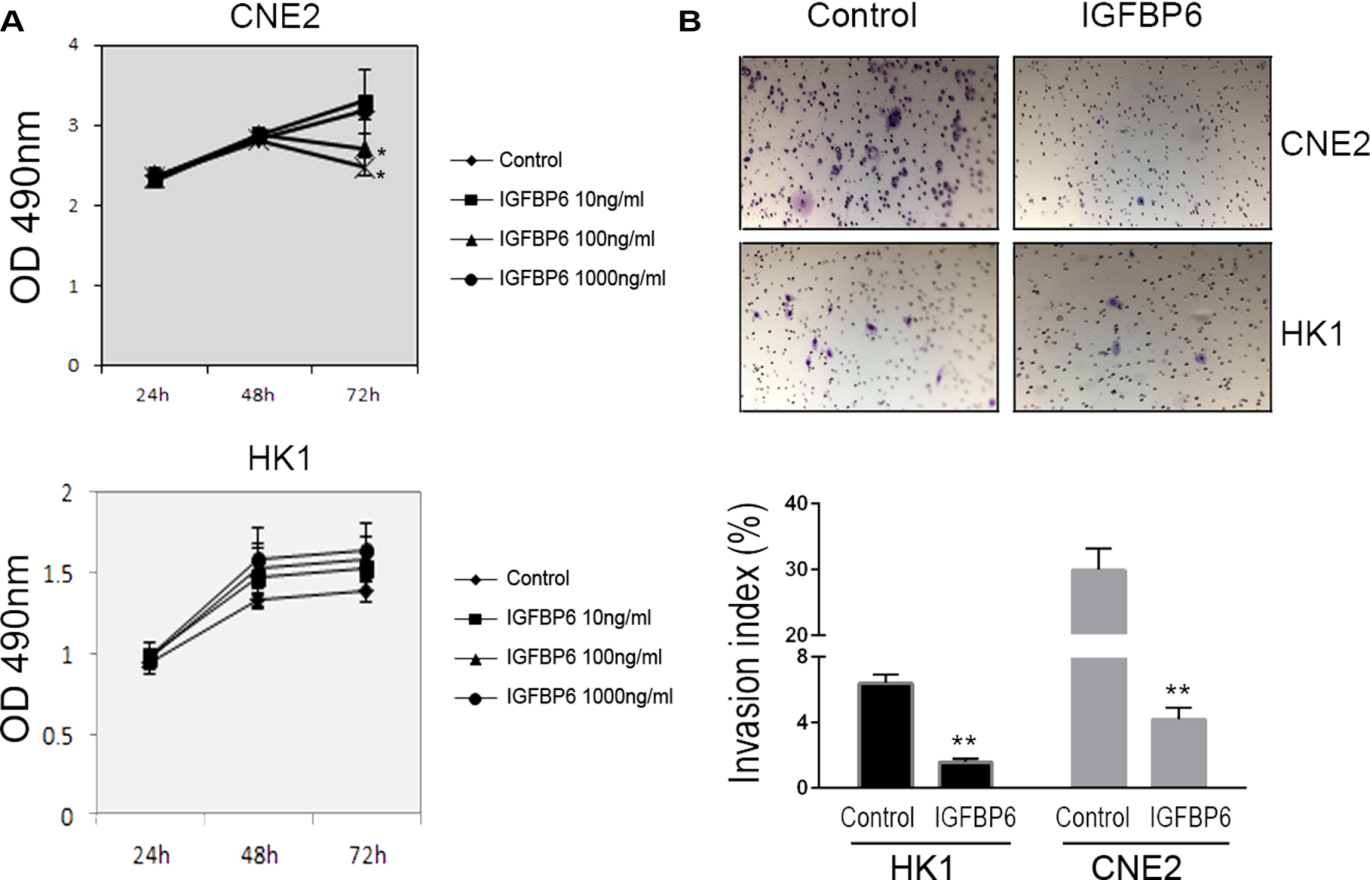
Recombinant human IGFBP6 decreases CNE2 cell proliferation and invasion CNE2 (upper panel) and HK1 (lower panel) cell proliferation as measured by MTS assay (**A**) Data represent means ± SD from six wells. *P < 0.05 compared to controls (IGFBP6 0 ng/ml). In transwell assays (upper panel), exogenous IGFBP6 inhibited CNE2 and HK1 cell invasion compared to controls (**B**) Invasive Index (%) was calculated (lower panel) according to the manufacturer's instructions. Columns, means of triplicate assays; bars, SE. **P < 0.01 compared to controls.

We investigated whether IGFBP6 influenced tumor cell proliferation and invasion *in vitro*. CNE2 and HK-1 cells were treated with the indicated concentrations of recombinant human IGFBP6 (rhIGFBP6) and cell proliferation was measured by MTS assay. Exogenous rhIGFBP6 dose-dependently inhibited CNE2 cell proliferation. The greatest inhibitory effect was achieved at 72 h with 100 ng/ml rhIGFBP-6 (Figure 3A). However, the inhibitory effect of IGFBP6 on HK1 cell proliferation was not significant. We then used a transwell assay to assess NPC cell invasion *in vitro*. CNE2 and HK1 cell invasion was reduced following rhIGFBP6 treatment (CNE2 86.2%, HK1 75.6%) compared to controls (Figure 3B).

### IGFBP6 knockdown promotes CNE2 cell proliferation and invasion through GSK3β/β-catenin/cyclin D1 pathway activation

**Figure 4:**
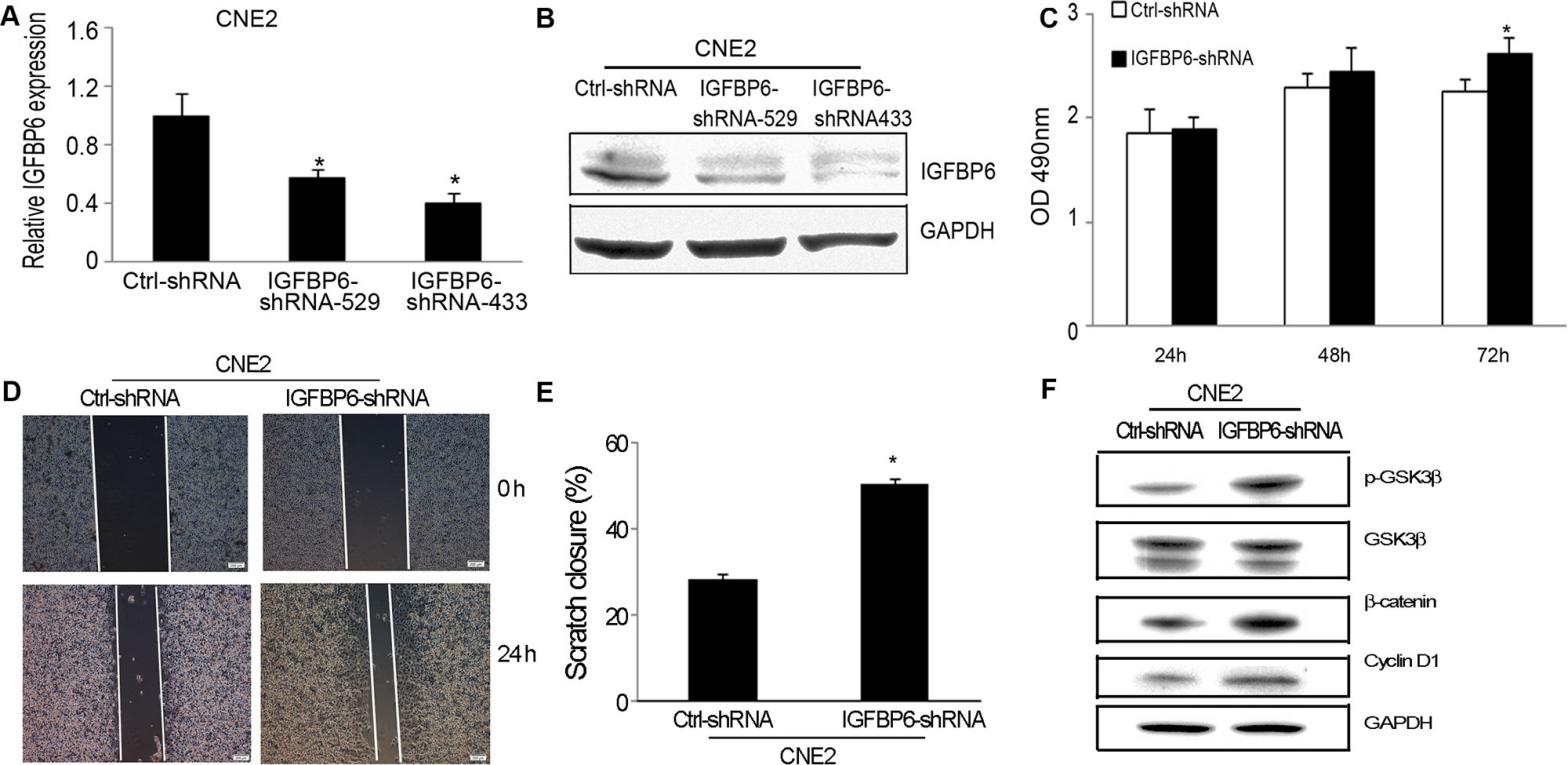
IGFBP6 knockdown in CNE2 cells promotes cell proliferation and invasion *in vitro* CNE2 cells were stably transfected with IGFBP6-shRNA. Real-time RT-PCR confirmed knockdown efficiency (**A**) Bars, ± SE. Data are representative of three separate experiments. Western blotting confirmed IGFBP6 knockdown (**B**) IGFBP6 knockdown induced tumor cell proliferation compared to controls (**C**) Representative wound-healing assay images (**D**) IGFBP6 knockdown increased tumor cell migration (**E**) Data represent means ± SD. *P < 0.05 compared to controls. Western blotting revealed GSK3β/β-catenin/cylin D1 pathway activation as a result of IGFBP6 knockdown (**F**).

We knocked down endogenous IGFBP6 in CNE2 cells to assess tumor cell growth, invasion and metastasis *in vitro* and *in vivo*. We produced two IGFBP6 knockdown lines, IGFBP6-shRNA-529 and IGFBP6-shRNA-433, and selected IGFBP6-shRNA-433 (IGFBP6-shRNA) for functional studies. IGFBP6 expression was unchanged in the scrambled control-transfected cell line (Ctrl-shRNA). IGFBP6 knockdown efficiency was confirmed by real-time PCR (Figure 4A) and western blotting (Figure 4B). IGFBP6 knockdown induced cell proliferation, especially at 72 h (Figure 4C). Wound-healing assays showed that IGFBP6 knockdown accelerated cell migration (Figure 4D–4E). Β-catenin is a tumor-associated transcriptional factor that promotes cell proliferation, migration and invasion. IGFBP6 knockdown increased GSK3β phosphorylation and cyclin D1 expression and induced β-catenin accumulation.

### IGFBP6 knockdown promotes CNE2 cell tumor growth and metastasis in mice

**Figure 5:**
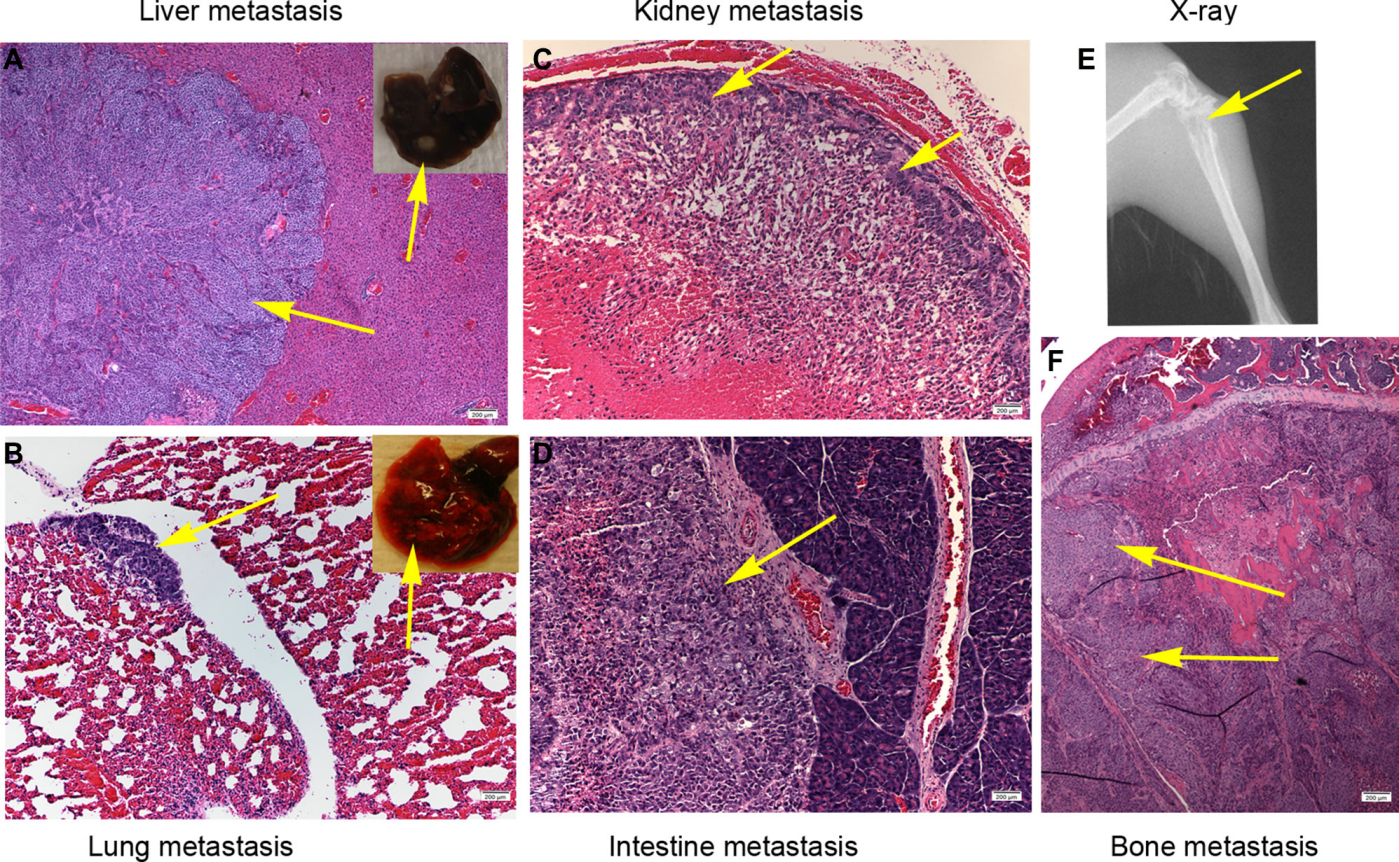
IGFBP6 knockdown in CNE2 cells enhances multiple distant organ metastases in mice Single-cell suspensions (5 × 10^5^ cells in 20 μL PBS) were injected into the left ventricles of SCID mice, and mice were sacrificed after three weeks. Representative micrographs of distant organ metastases *in vivo*. Liver (**A**) Lung (**B**) Kidney (**C**) Intestine (**D**) X-ray showing bone destruction (**E**) Bone metastasis (**F**) Yellow arrow points to tumor cells.

We injected CNE2 cells transfected with either ctrl-shRNA or IGFBP6-shRNA into the left ventricles of severe combined immunodeficiency (SCID) mice to assess tumor cell metastasis. Three weeks post-injection, mice were sacrificed and evidence of distant organ metastasis was evaluated histologically. IGFBP6 knockdown promoted metastasis: 15/15 mice in the IGFBP6-shRNA group, but only 5/15 in the Ctrl-shRNA group, showed evidence of metastasis (Table 3). Mice injected with IGFBP6-shRNA-transfected cells had higher metastasis rates, and tumor cells metastasized to multiple distant organs, such as liver, lung, bone, kidney and intestine (Figure 5).

**Table 3:** Silencing IGFBP6 expression in CNE2 cells promotes tumor metastasis in a mouse model

Cell line	Number of mice	Metastasis mice	Bone metastasis	Liver metastasis	Lung metastasis	Intestine metastasis	Kidney metastasis
Ctrl-shRNA	15	5	2	3	0	0	0
IGFBP-6-shRNA	15	15^*^	12	10	9	3	3

* *P* < 0.05.

## DISCUSSION

To date, radiation therapy is the main treatment strategy for NPC patients, and prognoses are mainly based on clinical stage. However, some patients with early stage disease experience locoregional relapse and distant metastasis shortly after treatment, while some advanced stage patients achieve long-term survival. This may be due to the biological heterogeneity of NPC. Therefore, novel prognostic markers and therapeutic targets are needed to guide and improve individual NPC patient treatment strategies.

Aberrant stimulation of the IGF axis can contribute to cancer development and progression [8]. However, few studies have provided insight into the effects of IGFs or IGFBPs on NPC. One report showed that EGF and IGF-I receptor levels were higher in several NPC cell lines as compared to normal nasopharyngeal epithelial cells [28]. Fung, *et al.* reported that IGF receptor II was overexpressed in malignant nasopharyngeal epithelial cells [29]. Only one *in vitro* study proposed that IGFBP6 is a tumor suppressor in NPC [27].

The current study found that IGFBP6 was differentially expressed in NPC tissues. Positive IGFBP6 expression was associated with reduced locoregional relapse and distant metastasis risk. Multivariate analysis in a Cox model showed that IGFBP6 was an independent prognostic biomarker for locoregional relapse and distant metastasis in NPC patients. Consistent with our findings, Zhang, *et al.* identified chemokine (C-C motif) ligand 18 (CCL18) and IGFBP6 as novel serum biomarkers for prostate cancer [30]. IGFBP6 levels increased after Diethylstilbestrol treatment, and recombinant IGFBP6 inhibited cell proliferation [18]. Serum IGFBP6 concentrations were lower in breast cancer patients as compared to those with benign breast disease [31]. These findings suggest that IGFBP-6 may serve as a marker in multiple cancers [32].

We also measured IGFBP6 in the NPC cell lines, CNE2, CNE1, SUNE1, HK1 and HONE1, all of which expressed IGFBP6 at different levels. We found that exogenous rhIGFBP6 decreased CNE2 cell proliferation and invasion *in vitro*. RhIGFBP6 reduced HK1 cell invasion, but had no effect on proliferation. IGFBP6 knockdown in CNE2 cells promoted tumor cell metastasis *in vivo*. These results implied that IGFBP6 expression suppresses NPC metastasis. Compared to controls, the GSK3β/β-catenin/cyclin D1 pathway was activated in CNE2 cells transfected with IGFBP6-shRNA. Cyclin D1 expression and GSK3β phosphorylation increased, and β-catenin accumulated in transfected cells. To the best of our knowledge, this study is the first to provide direct evidence that IGFBP6 inhibits NPC metastasis via GSK3β/β-catenin/cyclin D1 pathway activation.

Our results demonstrate that IGFBP6 may act as a novel prognostic biomarker in NPC. More aggressive treatments, such as targeted chemotherapy, may be advised in NPC patients negative for IGFBP6, who are more likely to experience distant metastasis.

## MATERIALS AND METHODS

### Reagents

Recombinant human IGFBP6 (rhIGFBP6) and the anti-human IGFBP6 monoclonal antibody were purchased from R&D Systems (Minneapolis, MN). All chemical reagents were purchased from Sigma (St. Louis, MO).

### Animals

SCID mice (Charles River, Wilmington, MA), 6 weeks of age, were housed under pathogen-free conditions in accordance with NIH guidelines. The animal protocol was approved by the Institutional Animal Care and Use Committee, University of Pittsburgh.

### Patient samples

Tumor tissues collected from 76 advanced NPC patients between September 1998 and December 2004 were obtained from the Sun Yat-sen University Cancer Center in Guangzhou, China. All NPC clinical diagnoses were confirmed by histopathologic examination. The University Institutional Review Board (IRB) approved the specimen collection and experimental protocols. Radiotherapy techniques and dose schedules were the same for all patients. Patients consented and were treated with a uniform radiotherapy protocol in line with the NPC treatment policy of Sun Yat-sen University Cancer Center.

### Patient follow-up and prognosis

After treatment completion, patients were followed up at least every three mo during the first three years and every six mo thereafter until death. Nasopharyngoscopy, MRI of the head and neck, chest radiography and abdominal sonography were routinely performed annually or at the time of tumor relapse.

### Cell lines and culture

Human NPC cell lines, CNE1, CNE2, HONE1, HK1 and SUNE1, were kindly provided by Dr. Chao-Nan Qian (Department of Nasopharyngeal Carcinoma, Cancer Center, Sun Yat-sen University, Guangzhou, China) and cultured in DMEM medium (Invitrogen, Carlsbad, CA). Media were supplemented with 10% fetal bovine serum (FBS, Invitrogen), 100 units/ml penicillin and 100 μg/ml streptomycin (Invitrogen). All cells were maintained in 10-cm tissue culture dishes in a 37°C incubator with 5% CO_2_ in humidified air.

### Conditioned media

Conditioned media (CM) was obtained from selected NPC cell cultures. Briefly, 2 × 10^6^ cells per dish were plated in 10-cm tissue culture dishes for 12 h in DMEM with 10% FBS. Media were then changed to 10 ml DMEM plus 1% FBS and supernatants were collected 48 h later. To normalize for cell density differences due proliferation during the culture period, cells from each plate were collected and total DNA content/plate was determined (spectrophotometric absorbance, 260 nm). CM was normalized for DNA content between samples by dilution with DMEM.

### IGFBP6 knockdown

Block-it RNAi designer (GenePharma) was used to design short hairpin RNA molecules (shRNA) specific to human IGFBP6 (Accession No: NM_002178; position 397–418; 5′-CAC CGC TGT TGC AGA GGA GAA TCC TCG AAA GGA TTC TCC TCT GCA ACA GC-3′; position UTR3; 5′-CAC CGC TGG TTG GAA AGA GTG TTG GCG AAC CAA CAC TCT TTC CAA CCA GC-3′). Control scrambled shRNA (ctrl-shRNA) was generated by inverting the bases at positions 9–13 within the IGFBP6 sequence. Resulting sequences were cloned into the RNA expression vector, pENTR/H1/TO (Invitrogen), and sequences were confirmed by sequencing (Invitrogen). IGFBP6-shRNA and ctrl-shRNA were separately transfected into CNE2 cells using lipofectamine reagents (Invitrogen) and individual clones were selected using 600 μg/ml G418 (Invitrogen). Two clones were used to evaluate IGFBP6 knockdown.

### Real-time RT-PCR

Real-time RT-PCR was performed in an iCycler iQ multicolor real-time PCR detection system (Bio-Rad, Hercules, CA) using the iScript one-step RT-PCR kit with SYBR Green (Bio-Rad). Primers used were as follows: IGFBP6: sense 5′-GAC CAG GAA AGA ATG TGA AAG TGA-3′, antisense 5′-GCT CTG CCA ATT GAC TTT CCT TAG-3′; GAPDH: sense 5′- CCA TGG AGA AGG CTG GGG-3′, antisense 5′- CAA AGT TGT CAT GGA TGA CCT −3′. The qRT-PCR method as as follows: 50°C for 10 min, denaturation at 95°C for 5 min, 45 cycles with denaturation at 95°C for 30 s, annealing at 55°C for 15 s and elongation at 72°C for 1 min. Fluorescence intensity of the double-strand-specific SYBR Green, reflecting the amount of formed PCR product, was monitored at the end of each elongation step. Melting curve analysis was performed to confirm PCR product purity. Quadruplicate samples were run for each primer set. IGFBP6 was normalized to GAPDH using the ΔCT method [33].

### Western blot analysis

Cell lysates were collected using standard procedures [34]. All samples were measured for total protein content by BCA assay (Pierce, Rockford, IL) to ensure equal loading. Loading buffer was added to 30 μg protein. Samples were boiled, resolved on 12% SDS-PAGE gels and then transferred onto PVDF membranes (Bio-Rad). Blots were blocked for 1 h at room temperature (RT) with shaking, then incubated overnight in primary antibody for IGFBP6 (1 μg/mL in blocking solution), p-GSK3β (Cell Signaling Inc.), GSK3β (Cell Signaling), β-catenin (Cell Signaling) or Cyclin D1 (R&D Systems) at 4°C with shaking. Blots were washed and incubated for 1 h with anti-mouse-IgG-HRP (1:2000). After washing, bands were detected using enhanced chemiluminescence (ECL) reagent (Amersham Biosciences, Piscataway, NJ) and exposed to light-sensitive film. GAPDH (Santa Cruz, CA) was detected as a loading control.

### Enzyme-linked immunosorbent assay (ELISA)

IGFBP6 in CM collected from selected NPC cell cultures was measured using an IGFBP6 ELISA kit (R&D Systems) following the manufacturer's protocol.

### Cell proliferation

Cell proliferation was measured using a CellTiter 96 AQeous Non-Radioactive Cell Proliferation Assay (Promega, Madison, WI). Briefly, cells were plated into 96-well plates at 3000 cells/well overnight. The next day, media was changed to 100 μL of DMEM plus 1% FBS. Indicated rhIGFBP6 concentration (0–1000 ng/mL) were added into the cultures. Cells were incubated at 37°C in a humidified 5% CO_2_ atmosphere for 24, 48 or 72 h, then 20 μL of combined MTS/PMS solution was added. After incubation for 2 h at 37°C, absorbance at 490 nm was recorded for each well using an ELISA plate reader. Data represent the average absorbance for quadruplicate wells.

### Transwell assay

Cells were seeded in 24-well Matrigel invasion chambers (BD Biosciences, Bedford, MA) and cell migration and invasion assays were performed as previously described [35]. Briefly, cells were cultured in serum-free medium for 24 h, then collected and seeded at 5 × 10^4^ in 0.5 mL DMEM containing 0.2% BSA into the upper compartment of wells. 0.75 mL DMEM containing 10% FBS was placed into the lower compartment. Recombinant IGFBP6 (100 ng/mL) was placed in the indicated wells. Transwell chambers were incubated for 24 h at 37°C with 5% CO_2_. Then, cells in the upper chamber were removed. Cells that had invaded through the Matrigel matrix membrane were stained with crystal violet after fixation with paraformaldehyde. Invaded cells were quantified by counting cells that penetrated the membrane in ten microscopic fields (at 200× magnification) per filter. Invasive index was defined as the proportion of cells that penetrated the Matrigel-coated membrane to the number of cells that migrated through the uncoated membrane.

### Wound-healing assay

Cells were seeded into 24-well tissue culture plates at 1 × 10^5^cells/well for 24 h. Then, the monolayer of each well was gently scratched with a 200-μL pipette tip across the center. The gap distance equaled the outer diameter of the end of the tip. In each well, the first straight-line scratch was crossed by a second straight-line scratch perpendicular to the first, creating a cross in each well. After scratching, wells were gently washed twice with medium to remove detached cells. Media were replenished and cells were allowed to grow another 24 h. Photos of each well were then taken under a microscope. Gap distances and areas were quantitatively evaluated using Image J software. Quadruplicate views of each well were documented, and each experimental group was repeated three times.

### Intracardiac injection of NPC cells into SCID mice

Single-cell suspensions of IGFBP6 knockdown CNE2 cells (IGFBP6-shRNA) or CNE2 cells with ctrl-shRNA (5 × 10^5^ cells in 20 μL PBS) were injected into the left ventricle of SCID mice (*n* = 15/group). Tumor cells were allowed to grow for three weeks, at which time mice were sacrificed. Evidence of distant organ tumor metastasis was evaluated histologically.

### Immunohistochemical staining

Tissue slides were stained following a modified protocol [36]. Briefly, slides were heated at 56°C for 45 min, deparaffinized and rehydrated, then immersed in 3% hydrogen peroxide for 10 min. Slides were incubated for 24 h at 4°C with anti-human IGFBP6 antibody (1:30 dilution) or isotype control mouse IgG2b (1:30 dilution). Biotinylated anti-mouse antibody (1:200) was used as the secondary antibody. After incubation in avidin-biotin complex solution, stain was developed using the diaminobenzidine method, followed by counterstaining with hematoxylin. Sections were analyzed using a Nikon NIS-Elements microscope. Immunostaining intensity was assessed independently by two clinical pathologists in a blinded manner. IGFBP6 protein staining was scored as negative (IGFBP6(−)) or positive (IGFBP6(+)) [37].

### Statistical analysis

Statistical analysis was performed using Statview software (Abacus Concepts, Berkley, CA). ANOVA was used for initial analyses, followed by Fisher's protected least significant difference for *post hoc* analyses. Five-year NPC patient survival rates were compared using the Kaplan-Meier method and analyzed by Log-rank test. Factors affecting survival rate were analyzed by Cox proportion hazard model using the SPSS statistic package Version 16.0. Differences with a *P* < 0.05 were considered statistically significant.
